# The Association Between Dietary Inflammatory Index with Metabolic Syndrome and Subclinical Atherosclerosis in Children and Adolescents: A Systematic Review

**DOI:** 10.5812/ijem-167139

**Published:** 2026-03-18

**Authors:** Parto Hadaegh, Amir Abdi, Farzad Hadaegh, Golaleh Asghari

**Affiliations:** 1 Prevention of Metabolic Disorders Research Center, Research Institute for Metabolic and Obesity Disorders, Research Institute for Endocrine Sciences, Shahid Beheshti University of Medical Sciences, Tehran, Iran; 2 Student Research Committee, Department of Clinical Nutrition and Dietetics, Faculty of Nutrition Science and Food Technology, Shahid Beheshti University of Medical Sciences, Tehran, Iran; 3 Student Research Committee, School of Medicine, Tehran Medical Sciences, Islamic Azad University, Tehran, Iran; 4 Department of Clinical Nutrition and Dietetics, Faculty of Nutrition Science and Food Technology, National Nutrition and Food Technology Research Institute, Shahid Beheshti University of Medical Sciences, Tehran, Iran

**Keywords:** Dietary Inflammatory Index, Metabolic Syndrome, Carotid Intima-Media Thickness, Cardiometabolic, Atherosclerosis, Adolescence

## Abstract

**Context:**

Metabolic syndrome (MetS) and increased carotid intima-media thickness (CIMT) in children and adolescents are critical early warning signs for the development of non-communicable disorders in adulthood. Identifying modifiable risk factors such as the Dietary Inflammatory Index (DII) is therefore essential for implementing early public health interventions to prevent irreversible long-term health consequences.

**Objectives:**

This systematic review aimed to evaluate the association of DII with MetS and CIMT in children and adolescents.

**Data Sources:**

Using three literature databases (PubMed, Web of Science, and Scopus), we searched for studies published between 1 January 2000 and 1 August 2024, which assessed the association of DII with MetS or CIMT among children and adolescents.

**Study Selection:**

Two reviewers screened and assessed the quality of studies using the Newcastle-Ottawa Scale.

**Data Extraction:**

Given the heterogeneity of study designs, a narrative synthesis approach was employed (PROSPERO: CRD420250643107).

**Results:**

Out of 23,393 records identified, two prospective cohorts and five cross-sectional studies met our inclusion criteria, including four and three studies focused on MetS and CIMT as the outcome, respectively. The included studies investigated a combined population of over 13,000 participants, comprising both sexes, aged 6 to 19 years. Two studies, one cross-sectional (odds ratio: 1.20; 95% confidence interval: 1.01, 1.43) and one prospective (β: 0.12; 0.003, 0.22), showed a harmful association between DII and MetS, while two other studies did not find any statistically significant association. Regarding the association between DII and CIMT, two studies noted null associations, while another study observed a positive association with high CIMT [odds ratio = 2.46 (1.15, 5.24)].

**Conclusions:**

In light of the inconsistent associations between the DII with MetS or CIMT reported in the literature, the suggestive evidence of harm from a small number of studies highlights a critical gap.

## 1. Context

Atherosclerotic cardiovascular disease (ASCVD) is the primary cause of cardiovascular disease (CVD) morbidity and mortality (1). The progression of ASCVD begins from childhood and develops for decades before the onset of cardiovascular complications (2, 3); therefore, it is valuable to detect early arterial changes in order to prevent the progression of CVD (4). The unfavorable trend in eating habits and dietary patterns in adolescents, along with the increasing trend of physical inactivity, has led to a progressive increase in the prevalence and incidence of metabolic syndrome (MetS) in this age group (5). MetS in children and adolescents is defined by the coexistence of abdominal obesity, dyslipidemia, elevated blood pressure, and impaired glucose metabolism, reflecting underlying insulin resistance (IR) (5, 6). These abnormalities often persist into adulthood and are independently linked to early vascular dysfunction and subclinical atherosclerosis, such as increased carotid intima–media thickness (CIMT) (7). Consequently, MetS in youth serves both as a marker of current cardiometabolic risk and as an intermediate stage in the life-course progression toward atherosclerotic cardiovascular disease (8). Because chronic, low-grade inflammation is a key mechanism linking obesity and IR to these vascular changes, the inflammatory potential of the diet is a plausible upstream determinant (9).

The Dietary Inflammatory Index (DII) was developed in 2014 to standardize the inflammatory potential of individuals’ diets (10). A total of 45 food parameters, including food items, macronutrients, micronutrients, and other constituents, create this index (10). The Children’s Dietary Inflammatory Index (C-DII) was developed based on the same schema and validated for use in pediatrics (11). Systematic reviews have examined the association between DII and health outcomes in adults, including CVD (12), cancers (13), pregnancy outcomes (13), and mental health (14). Moreover, in recent years, systematic studies and meta-analyses have investigated the relationship between DII and MetS in adults (15-17). On the other hand, studies about the association of DII and CIMT are also limited in pediatrics (18-20). A systematic review conducted by Suhett et al. investigated the role of dietary inflammatory potential on cardiometabolic risk among children and adolescents. Dietary patterns and components, as well as DII and C-DII, are accompanied by systemic inflammation (21); however, the investigators did not address the relationship of the exposure with MetS and CIMT.

## 2. Objectives

In the current study, we aimed to address the following question: “What is the evidence available regarding the association between the DII with MetS and CIMT in children and adolescents?”

## 3. Data Sources

The current systematic review was designed following the principles of the Preferred Reporting Items for Systematic Reviews and Meta-Analyses (PRISMA) statement guidelines (22) and was registered in the International Prospective Register of Systematic Reviews (PROSPERO) database on February 24, 2025 (CRD420250643107). The current study pertains to project NO. 1404/25123 from the Student Research Committee at Shahid Beheshti University of Medical Sciences in Tehran, Iran.

### 3.1. Search Strategy

The search was performed on the Web of Science, Scopus, and PubMed databases from January 2000 to August 2024. The following search terms were used with combinations of descriptors available at the Medical Subject Headings (MESH), non-MESH terms, and keywords related to the subject, which included the following terms: “dietary inflammatory index” OR “children dietary inflammatory index” AND “carotid artery intima-media thickness” AND “metabolic syndrome” AND “cardiometabolic syndrome” (Table 1 in Supplementary File). 

## 4. Study Selection

### 4.1. Eligibility Criteria (Population, Exposure, Comparison, and Outcome)

The population of interest comprised children and adolescents, typically aged 6 - 19 years, for evaluation of the main exposures. Exposure included the DII or C-DII in any version. Comparators were defined as lower versus higher DII/C-DII categories or per-unit/standard deviation increase. The primary outcomes were MetS, using any pediatric definition, as well as subclinical atherosclerosis measured by CIMT or other vascular markers if reported. Eligible study designs were observational studies, including cross-sectional, case–control, and cohort studies, with no geographical or language restrictions. Studies were excluded if they focused on adults, used non-DII dietary scores, reported non-original data, were reviews or conference abstracts without data, were animal or in vitro studies, or were conducted on special populations, e.g., cancer patients.

## 5. Data Extraction

### 5.1. Quality Assessment

The quality and risk of bias of the included studies were evaluated using the Newcastle–Ottawa Scale (NOS), a validated tool for assessing non-randomized studies (23). To evaluate the quality of included cohort studies, NOS, and for cross-sectional studies, an adapted version of NOS was used (24). This scale consists of three key domains—selection, comparability, and outcome, comprising a total of eight items. Each study was independently assessed by two reviewers (P.H. and A.A.), with disagreements resolved through discussion with a third reviewer (G.A.). The NOS assigns a score ranging from 0 to 10, with studies scored 7 - 10 considered to have a low risk of bias, while those scoring 4–6 indicated an adequate risk, and scores of 0 - 3 reflected a high risk of bias.

### 5.2. Data Synthesis

Two independent reviewers (P.H. and A.A.) screened and reviewed the titles and abstracts of relevant studies and performed the study selection, whereas a chief investigator (G.A.) was present to resolve any disagreements. Studies relevant to inclusion were identified by reviewing the full text of the potentially eligible articles.

Because of substantial methodological heterogeneity across studies (different DII/C-DII versions, dietary assessment tools, cut-points for DII, multiple pediatric MetS definitions, and variable reporting of subclinical atherosclerosis), we did not undertake a quantitative meta-analysis. Instead, we performed a narrative synthesis. Studies were first grouped by outcome: (A) MetS, and (B) subclinical atherosclerosis. From each study, the following data were recorded: first author, year of publication, country, WHO region [Eastern Mediterranean Region (EMR), Region of the Americas (AMR), European Region (EUR), South-East Asia Region (SEAR), Western Pacific Region (WPR)], study design and setting, population characteristics, dietary assessment method, MetS definition, DII/C-DII operationalization (continuous vs categorical), statistical model and covariate adjustment (particularly adiposity and energy intake), and direction and magnitude of the reported association. Where studies reported multiple models, we described the most fully adjusted model. We highlighted inconsistencies and potential sources of heterogeneity (age range, sex distribution, region, outcome definition) rather than producing pooled effect estimates.

## 6. Results

### 6.1. Description of Studies

**Figure 1.**
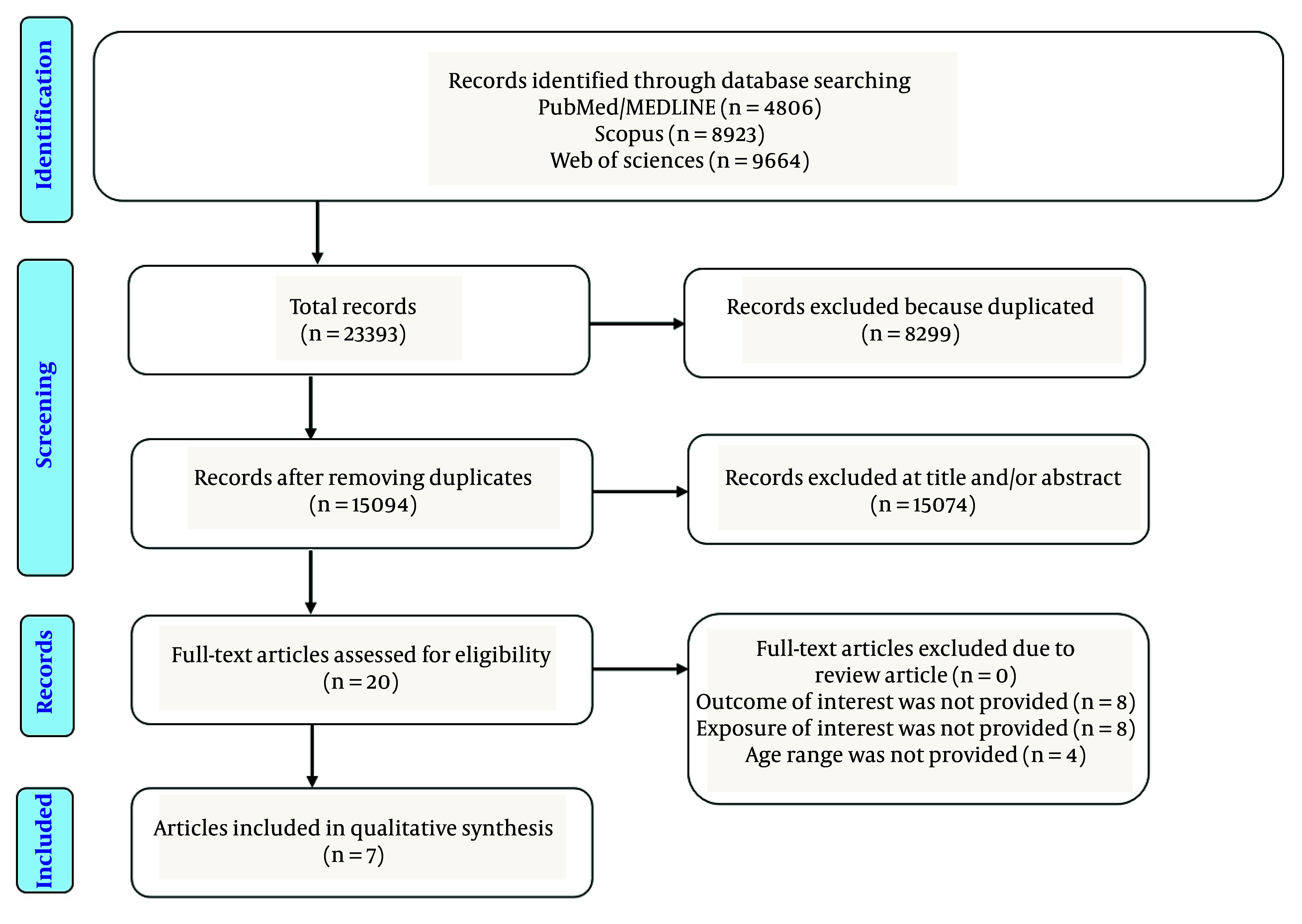
Flow chart of included studies

A total of 23,393 references from databases and manual searches published from 2000 to 2024 were identified. After removing duplicate articles (n = 8,299), a total of 15,094 articles were screened using the title and abstract. The articles were investigated after full screening based on inclusion and exclusion criteria. Eventually, 2 cohort and 5 cross-sectional studies were found to be eligible for inclusion in the systematic review (Figure 1). The included studies examined a total population of 13,202 participants.

The characteristics of the articles included, such as author and year of publication, location and WHO region, design of the study and study period, sample size and proportion of females, age range, body mass index (BMI), inflammatory score range, dietary assessment method, MetS definition, exposure, outcome, covariates, and results are described in Table 1. The number of participants ranged from 100 to 5,656, with an age range between 1 and 24 years. All the studies reported both genders in combination. Of the studies, one originated from the EMR, three from the AMR, two from the EUR, and one from the WPR. Considering the dietary assessment method, four studies used the Food Frequency Questionnaire (FFQ), two studies used dietary records, and one study used 24-hour dietary recalls. Of the total of seven articles, four were with the outcome of MetS, and three were with the outcome of CIMT. Out of the four articles with the MetS outcome, three used the International Diabetes Federation (IDF) for defining MetS, and one used both IDF and Adult Treatment Panel III (ATP).

**Table 1.** Detailed Characteristics of Studies on the Associations of Dietary Inflammatory Index with Metabolic Syndrome and Carotid Intima-Media Thickness in Children and Adolescents

Author (y)	Country/WHO Region	Design (Study Period)	Sample Size (Female%)	Age Range (y)	BMI	Inflammatory Score Range	Dietary Assessment Method	Exposure	Outcome (MetS Definition)	Covariates	Results
**Davis et al. (2019) (** 18 **)**	Australia/WPR	Cross-sectional	1771 (49%)	11 - 12	NA	-6 to +29	FFQ	Literature-derived inflammatory diet score and - GlycA- derived inflammatory diet score	CIMT	Age, sex, SEP, pubertal status, BMI z-score, atrial diameter	No significant association was shown for vascular structure and function markers as the outcome
**Rahbarinejad, et al. (2019) (** 19 **)**	Iran/EMR	Cross-sectional	339 (48%)	6 - 13 ^a^	23.1 (SD 3.1)	-5.43 to +4.41	FFQ	DII	CIMT	Age, sex, puberty status, physical activity, total energy, fruits, vegetables, body fat percentage	The odds of having CIMT ≥ 0.43mm were reported to be 2.46 times higher among those with DII scores in the highest quartile compared to those in the lowest quartile. This finding highlights the CIMT ≥ 0.43mm cutoff point, which could be interpreted as suggesting that pro-inflammatory diets may exacerbate the risk of CIMT ≥ 0.43mm.
**Betanzos-Robledo, et al. (2020) (** 25 **)**	Mexico/AMR	Prospective (22 years)	100 (46%)	1 - 22	Median: 24.3 [IQR: 21.9 -27.3]	-2.46 to +4.71	FFQ	C-DII; DII	MetS z-score (IDF)	Sex, socioeconomic status, smoking status, physical activity, birth weight, BMI	Positive association between cumulative DII and MetRisk (beta regression coefficient for the association between the AUC of DII and the MetRisk Z-score); 0.12 (0.03, 0.22), P < 0.009
**Seremet Kurklu, et al. (2020) (** 26 **)**	Turkey/EUR	Cross-sectional	343 (63%)	10 - 16	NA	+1.04 to +5.11	Food records	DII	MetS (IDF)	Age, sex, BMI, physical; Activity, energy intake	A pro-inflammatory diet was associated with increased odds of MetS in Q4 vs. Q1; crude OR: 3.66 (1.27 - 10.49); Adjusted OR: 2.60 (0.82 - 8.28)
**Wang, et al. (2022) (** 27 **)**	Ecuador/AMR	Cross-sectional	276 (55.4%)	6 - 12	17.2 (SD 2.7)	-4.87 to +4.75	FFQ	DII	MetS (IDF)	Age, sex, ethnicity, neighborhood, maternal; Education, household per capita income, BMI	By a one-unit increase in DII score, MetS increased by 1.20 (1.01 - 1.43) for both crude and adjusted models.
**Jia, et al. (2022) (** 28 **)**	United States/AMR	Cross-sectional	5656 (48.23%)	12 - 19	23.8 (SE 0.11)	-1.95 to -0.79	24-h food recall	DII	MetS (IDF and ATP)	Age, gender, race, family income to poverty ratio, education level, smoke exposure, and physical activity level	Q4 vs. Q1; Crude OR: 1.31 (0.93 - 1.85); Adjusted OR: 1.09 (0.73 - 1.62)
**Buckland, et al. (2024) (** 20 **)**	United Kingdom/EUR	Prospective (17 years)	4717 (50.7%)	7 - 24	At 7 years: 16.1; At 10 years: 18.0; At 13 years: 20.3	-	Food records	C-DIS	CIMT	Sex, plausibility of dietary reporting, maternal highest education attainment, family's highest social class, moderate-to-vigorous physical activity level, puberty timing	No significant association was shown for CIMT as the outcome.

Abbreviations: MetS, metabolic syndrome; WPR, Western Pacific Region; NA, not available; FFQ, Food Frequency Questionnaire; CIMT, carotid intima-media thickness; SEP, socioeconomic position; BMI, Body Mass Index; EMR, Eastern Mediterranean Region; DII, Dietary Inflammatory Index; AMR, region of the Americas; C-DII, Children’s Dietary Inflammatory Index; IDF, International Diabetes Federation; ATP, adult treatment panel III; EUR: European region; C-DIS, dietary inflammatory score adapted for children.

^a^ This study was conducted on overweight and obese children and adolescents.

### 6.2. Risk of Bias Assessment

According to the NOS for quality assessment of studies, two studies scored 7 points, two studies scored 8 points, and three studies scored 9 points, and the methodological quality of all seven studies was high (Table 2 in Supplementary File). 

### 6.3. Findings from the Systematic Review

Among four studies with MetS outcome (25-28), two studies found a positive association between DII and MetS. In the cohort of the early life exposures in Mexico to environmental toxicants (ELEMENT), a cumulative pro-inflammatory diet in childhood and adolescence was associated with increased MetS risk score, systolic blood pressure (SBP), and diastolic blood pressure (DBP) (25). Additionally, in a cross-sectional study conducted in Ecuador, with every one-unit increase in DII score, MetS increased by 1.20 in the covariate-adjusted model (27).

Of the three studies with CIMT outcome (18-20), just one study found a positive association between DII and CIMT. The findings of the study show that the probability of having CIMT ≥ 0.43 mm among people who had a DII score in the highest quartile is 2.46 times higher than those who were in the lowest quartile; this finding highlights the cut point of CIMT ≥ 0.43 mm (19). Davis et al. conducted a study in Australia within 1,771 participants (49% girls), and found no significant association between a pro-inflammatory diet and preclinical cardiovascular phenotypes in childhood (18). In the Avon longitudinal study of parents and children (ALSPAC), which included 4,717 participants (50.7% girls) with 17 years of follow-up, a low C-DIS Z-score at 10 years was associated with a lower pulse wave velocity (PWV) at 17 years; also, no significant correlation was observed between C-DIS Z-score and CIMT (20).

## 7. Conclusions

In this review, for the first time, we investigated the association of DII, as a proxy for the inflammatory entity of the diet, with MetS and CIMT among children and adolescents. Accordingly, we found a signal for the harmful effects of DII for MetS among two out of four studies. In terms of subclinical atherosclerosis as assessed by CIMT, this was found only in one study conducted among overweight and obese Iranian pediatrics.

Using 45 dietary components, DII was presented as a tool to assess the inflammatory potential of a diet (10); hence, it was expected that higher DII would be associated with cardiometabolic disorders, cancer, and mortality events (15, 29-31). Meta-analysis in adults, using both cross-sectional and prospective studies, found higher DII was associated with more than 20% higher risk for MetS (17). However, no significant association was found between the DII and the risk for MetS in another meta-analysis conducted among adult populations (15). The different results in studies examining the association between DII and MetS might be explained by important factors such as different methods for dietary assessment (FFQ vs dietary records vs 24-hour dietary recalls), variation in the number of food items to estimate DII, sample size, age range of study population, MetS definition (IDF vs ATP), and level of adjustment for confounders; the factors that applied to studies conducted in both adults as well as children and adolescents; the issue that was also addressed in the current study (Table 1). 

In our review, we summarize the findings from three cross-sectional studies that examined the association of DII with MetS and its components. Seremet Kurklu et al., among 343 Turkish adolescents aged 10 - 16 years, assessed the DII-MetS relationship, using DII scores calculated based on three consecutive days of food records, and found that the fourth quartile of DII was accompanied by an odds ratio of 2.6, but this value did not reach significance due to the limited number of events (95% CI: 0.82 - 8.28). Moreover, a more pro-inflammatory diet was associated with higher fasting plasma glucose (FPG) and triglyceride (TG) levels (26). In another study conducted among school-age Latin American (6 - 12 years), Wang et al. noted a one-unit increment was associated with a 20% odds ratio for prevalent MetS; the associations were mainly attributable to a strong relationship between DII, TG, and DBP (27). Jia et al. leveraged data from the National Health and Nutrition Examination Survey (NHANES) database 2001 - 2018, using DII calculated from 24-hour dietary recall to determine its association with MetS and its components, and found that high DII is strongly associated with prevalent high blood pressure (odds ratio: 2.27, 95% CI: 1.02 - 5.07) but not MetS (28).

Regarding prospective studies, the first study was conducted by Betanzos-Robledo et al., who examined the association between the inflammatory status of diets collected from infancy with MetS among young Mexican adults. They found that cumulative exposure of DII as assessed by area under the curve (AUC of DII) was positively associated with MetS z-score; the relationship was mainly explained by the strong association of DII with SBP and DBP (25). To our literature review, no other studies directly examined the association between DII/C-DII with MetS; however, other studies assessed this relationship for insulin resistance (IR). For example, using repeated measurements of diet quality and cardiometabolic risk (CMR) among Mexican children and adolescents aged 8 - 14 years, the higher C-DII was not associated with log HOMA-IR even in the crude model; however, among different components of MetS, the significant association was demonstrated for log TG in the multivariate analysis (32). In a British birth cohort study, ALSPAC, the investigators found that higher C-DIS at 7 years was significantly associated with higher cardiometabolic risk score [estimated using mean sex-specific z-scores from TG, HDL-C, low-density lipoprotein cholesterol (LDL-C), mean arterial blood pressure, HOMA-IR, and fat mass index] at both 17 and 24 years. The investigators concluded that a more pro-inflammatory diet during childhood was accompanied by an unfavorable cardiometabolic profile in adolescents/early adulthood (33).

As another aim in our review, we assessed the relationship between the inflammatory potential of the diet with subclinical atherosclerosis as assessed by CIMT. Using two inflammatory diet scores (literature-derived inflammatory diet score and GlycA-derived inflammatory diet score) other than the DII score, Davis et al., among 11 - 12-year-old Australian children, observed no trace of association between inflammatory scores and CIMT. Reporting a negative regression coefficient for the association between Literature-derived inflammatory diet score and CIMT, while noting a positive relationship for the GlycA-derived inflammatory diet score; however, none of these reached statistical significance (18). In another cross-sectional study among Iranian children and adolescents, the authors found that an increasing value of the DII score was associated with high CIMT (≥ 0.43 mm) in the multivariate analysis (19). Lastly, in the ALSPAC study, no association was found between increasing value of C-DIS score at three age stages of 7, 10, and 13 years with CIMT at both 17 and 24 years (20).

It was shown that systemic immune–inflammation index and systemic inflammatory response index can be used for the early diagnosis of MetS in pediatric obesity patients (34). Moreover, chronic inflammation, driven by poor dietary patterns, plays an important role in increasing blood pressure through endothelial dysfunction, arterial stiffness, and oxidative stress, all of which contribute to early vascular damage and elevated cardiovascular risk (35, 36). Similarly, an inflammatory dietary profile contributes to increased inflammatory cytokine production, oxidative stress, and lipid metabolism disorders, ultimately raising TG levels (37).

Some of the strengths of the present study are the systematic and comprehensive search within important literature databases, screening and quality assessment independently by two reviewers, and the study quality assessment by NOS. There are a number of limitations in the current review. First, the included studies exhibited heterogeneity in terms of study design, sample size, MetS definition, and geographic distribution. Second, there was a variation in dietary assessment methods and DII calculation across studies, as different food frequency questionnaires and dietary recall methods may yield inconsistent DII scores. Therefore, due to the heterogeneity of the extracted data, a meta-analysis was not feasible. Third, the majority of studies utilized a cross-sectional design, limiting the ability to establish causal relationships between DII and MetS or CIMT.

In conclusion, we found an important link between the DII and MetS; however, findings regarding this relationship between DII and subclinical atherosclerosis are very limited. Future reviews should emphasize the association between DII and MetS components rather than MetS or CIMT per se, especially high blood pressure and dyslipidemia, as found in the included studies.

## supplementary material

ijem-24-3-167139-s001.pdf

## Data Availability

The dataset presented in the study is available on request from the corresponding author during submission or after publication.
