# Supplementary material for: The Association Between Dietary Inflammatory Index with Metabolic Syndrome and Subclinical Atherosclerosis in Children and Adolescents: A Systematic Review
Source: Int J Endocrinol Metab. 2026 Mar 18;24(3):e167139. doi: 10.5812/ijem-167139 (PMC13181956; doi:10.5812/ijem-167139)
Supplement: ijem-24-3-167139-s001.pdf [file ijem-24-3-167139-s001.pdf]

Supplementary Table 1. Search strategy to identify observational studies reporting the associations of DII with MetS and CIMT

Database	Search terms	n
**PubMed**	((diet*[tiab] AND inflamm*[tiab]) OR “dietary inflammatory index”[tiab] OR DII[tiab] OR E-DII[tiab] OR “Children's Dietary Inflammatory Index”[tiab] OR C-DII[tiab] OR “inflammatory diet*”[tiab] OR “pro-inflammatory diet”[tiab] OR “proinflammatory diet”[tiab] OR “dietary inflammatory score”[tiab] OR DIS[tiab] OR (“inflammatory potential”[tiab] AND intake[tiab]) OR “inflammatory potential of diet”[tiab] OR “diet-related inflammation”[tiab] OR “dietary inflammatory potential”[tiab] OR "Empirical Dietary Inflammatory Index”[tiab] OR EDII[tiab] OR “empirical dietary inflammatory pattern”[tiab] OR EDIP[tiab] OR “anti-inflammatory diet”[tiab] OR “Anti-Inflammatory Diet Index”[tiab] OR “inflammatory score of the diet”[tiab] OR ISD[tiab]) AND ("Carotid Intima-Media Thickness"[Mesh] OR “intima media thickness”[tiab] OR “intima-media thickness”[tiab] OR “carotid intima media”[tiab] OR “artery intima media”[tiab] OR “artery wall thickness”[tiab] OR CIMT[tiab] OR IMT[tiab] OR “Carotid atherosclerosis”[tiab] OR “metabolic syndrome”[MeSH Terms] OR “metabolic syndrome”[tiab] OR “Mets”[tiab] OR “metabolic X syndrome”[tiab] OR “metabolic cardiovascular syndrome”[tiab] OR “reaven syndrome X”[tiab] OR “cardiometabolic syndrome”[tiab] OR “metabolic dysfunction”[tiab] OR “metabolic profile”[tiab] OR “insulin resistance syndrome”[tiab] OR “dysmetabolic syndrome”[tiab] OR “cardiometabolic syndrome”[tiab]) AND 2000/01/01:2024/08/31[Date - Publication]	4806
**Scopus**	TITLE-ABS-KEY((diet* AND inflamm*) OR “dietary inflammatory index” OR DII OR E-DII OR “Children's Dietary Inflammatory Index” OR C-DII OR “inflammatory diet*” OR “pro-inflammatory diet” OR “proinflammatory diet” OR “dietary inflammatory score” OR DIS OR (“inflammatory potential” AND intake) OR “inflammatory potential of diet” OR “diet-related inflammation” OR “dietary inflammatory potential” OR "Empirical Dietary Inflammatory Index” OR EDII OR “empirical dietary inflammatory pattern” OR EDIP OR “anti-inflammatory diet” OR “Anti-Inflammatory Diet Index” OR “inflammatory score of the diet” OR ISD) AND TITLE-ABS-KEY("Carotid Intima-Media Thickness" OR “intima media thickness” OR “intima-media thickness” OR “carotid intima media” OR “artery intima media” OR “artery wall thickness” OR CIMT OR IMT OR “Carotid atherosclerosis” OR “metabolic syndrome” OR “Mets” OR “metabolic X syndrome” OR “metabolic cardiovascular syndrome” OR “reaven syndrome X” OR “cardiometabolic syndrome” OR “metabolic dysfunction” OR “metabolic profile” OR “insulin resistance syndrome” OR “dysmetabolic syndrome” OR “cardiometabolic syndrome”) AND ((PUBYEAR > 1999 AND PUBYEAR < 2024) OR PUBDATETXT ("January 2024") OR PUBDATETXT ("February 2024") OR PUBDATETXT ("March 2024") OR PUBDATETXT ("April 2024") OR PUBDATETXT ("May 2024") OR PUBDATETXT ("June 2024") OR PUBDATETXT ("July 2024") OR PUBDATETXT ("August 2024"))	9664
**Web of Science**	TS=((diet* AND inflamm*) OR “dietary inflammatory index” OR DII OR E-DII OR “Children's Dietary Inflammatory Index” OR C-DII OR “inflammatory diet*” OR “pro-inflammatory diet” OR “proinflammatory diet” OR “dietary inflammatory score” OR DIS OR (“inflammatory potential” AND intake) OR “inflammatory potential of diet” OR “diet-related inflammation” OR “dietary inflammatory potential” OR "Empirical Dietary Inflammatory Index” OR edit OR “empirical dietary inflammatory pattern” OR	8923

	EDIP OR “anti-inflammatory diet” OR “Anti-Inflammatory Diet Index” OR “inflammatory score of the diet” OR ISD) AND TS=("Carotid Intima-Media Thickness" OR “intima media thickness” OR “intima-media thickness” OR “carotid intima media” OR “artery intima media” OR “artery wall thickness” OR CIMT OR IMT OR “Carotid atherosclerosis” OR “metabolic syndrome” OR “Mets” OR “metabolic X syndrome” OR “metabolic cardiovascular syndrome” OR “reaven syndrome X” OR “cardiometabolic syndrome” OR “metabolic dysfunction” OR “metabolic profile” OR “insulin resistance syndrome” OR “dysmetabolic syndrome” OR “cardiometabolic syndrome”) AND PY=(2000-2024)	
**All**	**-**	23393

**Supplementary Table 2.** Summary of quality assessment of included studies (*Newcastle-Ottawa scale*)				
**Cross-sectional studies (n=5)**				
**First Author (year)**	**Selection**	**Comparability**	**Outcome**	**Quality Score**
Davis, et al. (2019)	****	**	***	9/10
Rahbarinejad, et al. (2019)	****	**	***	9/10
Kurklu, et al. (2020)	***	**	***	8/10
Wang, et al. (2022)	***	**	***	8/10
Jia, et al. (2022)	****	**	***	9/10
**Cohort studies (n=2)**				
**First Author (year)**	**Selection**	**Comparability**	**Outcome**	**Quality Score**
Betanzos-Robledo, et al. (2020)	**	**	***	7/10
Buckland, et al. (2024)	**	**	***	7/10
